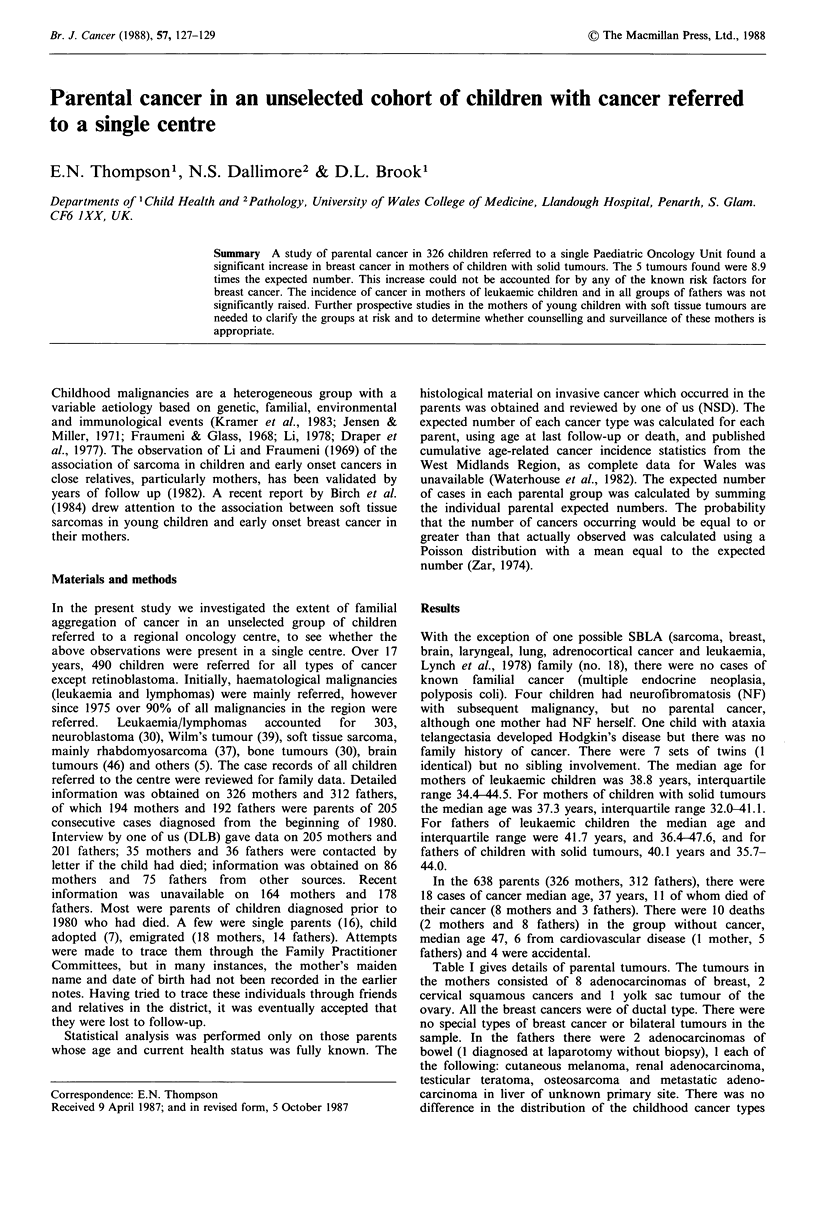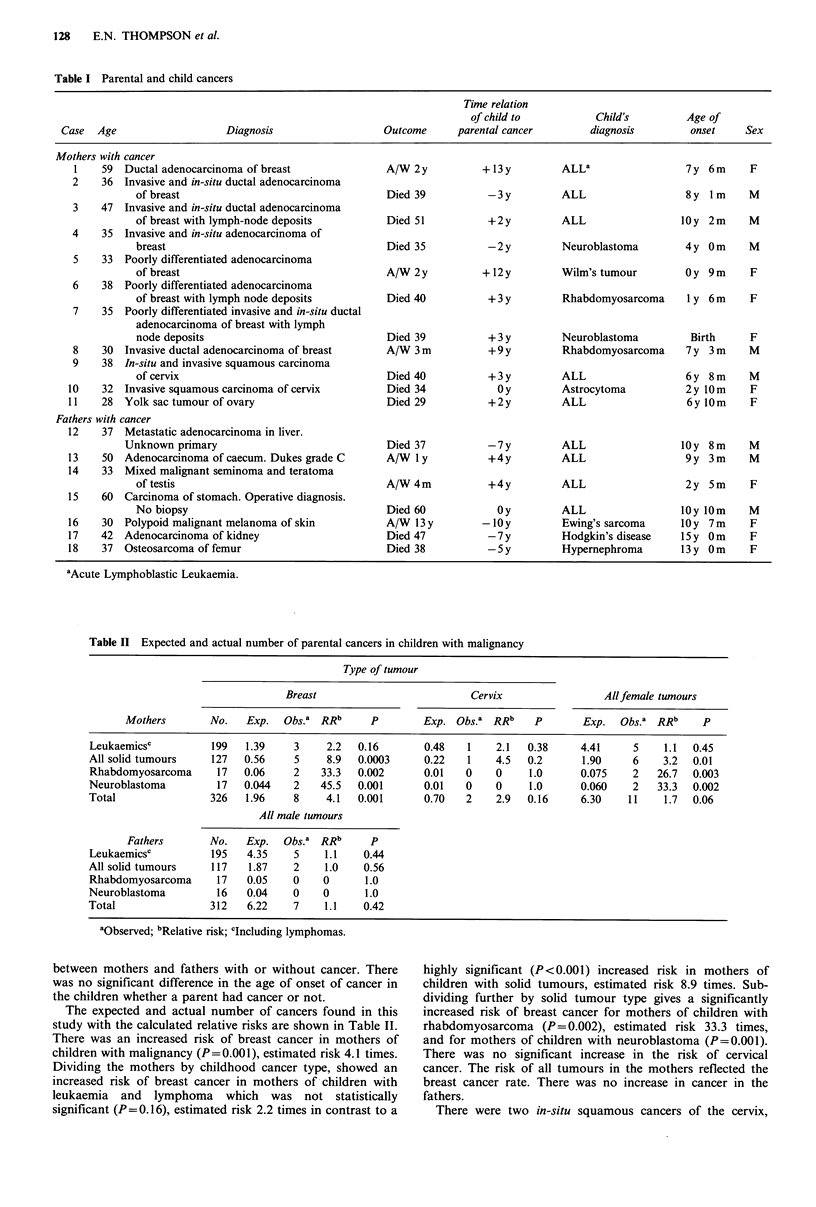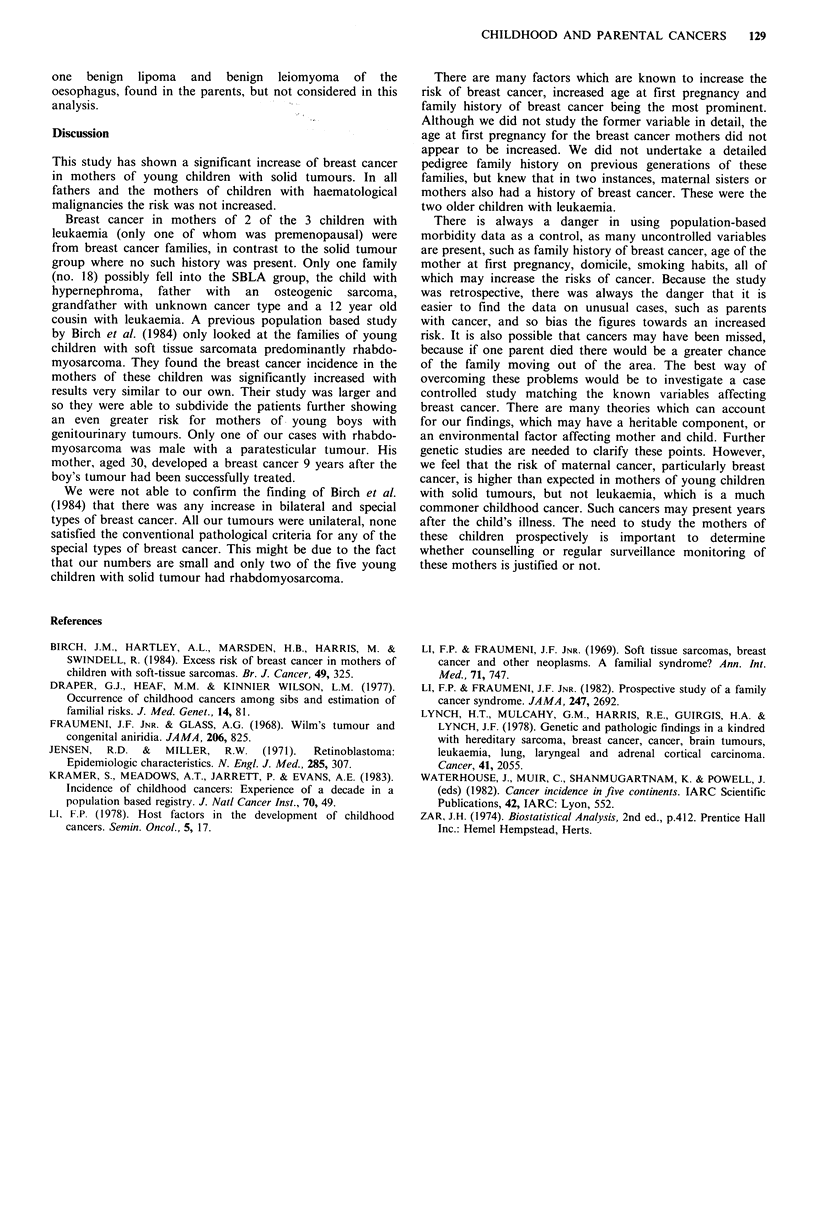# Parental cancer in an unselected cohort of children with cancer referred to a single centre.

**DOI:** 10.1038/bjc.1988.25

**Published:** 1988-01

**Authors:** E. N. Thompson, N. S. Dallimore, D. L. Brook

**Affiliations:** Department of Child Health, University of Wales College of Medicine, Llandough Hospital, Penarth, S. Glam., UK.

## Abstract

A study of parental cancer in 326 children referred to a single Paediatric Oncology Unit found a significant increase in breast cancer in mothers of children with solid tumours. The 5 tumours found were 8.9 times the expected number. This increase could not be accounted for by any of the known risk factors for breast cancer. The incidence of cancer in mothers of leukaemic children and in all groups of fathers was not significantly raised. Further prospective studies in the mothers of young children with soft tissue tumours are needed to clarify the groups at risk and to determine whether counselling and surveillance of these mothers is appropriate.


Br. J. Cancer (1988), 57, 127–129 © The Macmillan Press, Ltd., 1988

### Parental cancer in an unselected cohort of children with cancer referred to a single centre

E.N. Thompson[1], N.S. Dallimore[2] & D.L. Brook[1]

*Departments of [1]Child Health and [2]Pathology, University of Wales College of Medicine, Llandough Hospital, Penarth, S. Glam. CF6 1XX, UK.*

**Summary** A study of parental cancer in 326 children referred to a single Paediatric Oncology Unit found a significant increase in breast cancer in mothers of children with solid tumours. The 5 tumours found were 8.9 times the expected number. This increase could not be accounted for by any of the known risk factors for breast cancer. The incidence of cancer in mothers of leukaemic children and in all groups of fathers was not significantly raised. Further prospective studies in the mothers of young children with soft tissue tumours are needed to clarify the groups at risk and to determine whether counselling and surveillance of these mothers is appropriate.

Childhood malignancies are a heterogeneous group with a variable aetiology based on genetic, familial, environmental and immunological events (Kramer *et al.*, 1983; Jensen & Miller, 1971; Fraumeni & Glass, 1968; Li, 1978; Draper *et al.*, 1977). The observation of Li and Fraumeni (1969) of the association of sarcoma in children and early onset cancers in close relatives, particularly mothers, has been validated by years of follow up (1982). A recent report by Birch *et al.* (1984) drew attention to the association between soft tissue sarcomas in young children and early onset breast cancer in their mothers.

### Materials and methods

In the present study we investigated the extent of familial aggregation of cancer in an unselected group of children referred to a regional oncology centre, to see whether the above observations were present in a single centre. Over 17 years, 490 children were referred for all types of cancer except retinoblastoma. Initially, haematological malignancies (leukaemia and lymphomas) were mainly referred, however since 1975 over 90% of all malignancies in the region were referred. Leukaemia/lymphomas accounted for 303, neuroblastoma (30), Wilm's tumour (39), soft tissue sarcoma, mainly rhabdomyosarcoma (37), bone tumours (30), brain tumours (46) and others (5). The case records of all children referred to the centre were reviewed for family data. Detailed information was obtained on 326 mothers and 312 fathers, of which 194 mothers and 192 fathers were parents of 205 consecutive cases diagnosed from the beginning of 1980. Interview by one of us (DLB) gave data on 205 mothers and 201 fathers; 35 mothers and 36 fathers were contacted by letter if the child had died; information was obtained on 86 mothers and 75 fathers from other sources. Recent information was unavailable on 164 mothers and 178 fathers. Most were parents of children diagnosed prior to 1980 who had died. A few were single parents (16), child adopted (7), emigrated (18 mothers, 14 fathers). Attempts were made to trace them through the Family Practitioner Committees, but in many instances, the mother's maiden name and date of birth had not been recorded in the earlier notes. Having tried to trace these individuals through friends and relatives in the district, it was eventually accepted that they were lost to follow-up.

Statistical analysis was performed only on those parents whose age and current health status was fully known. The histological material on invasive cancer which occurred in the parents was obtained and reviewed by one of us (NSD). The expected number of each cancer type was calculated for each parent, using age at last follow-up or death, and published cumulative age-related cancer incidence statistics from the West Midlands Region, as complete data for Wales was unavailable (Waterhouse *et al.*, 1982). The expected number of cases in each parental group was calculated by summing the individual parental expected numbers. The probability that the number of cancers occurring would be equal to or greater than that actually observed was calculated using a Poisson distribution with a mean equal to the expected number (Zar, 1974).

### Results

With the exception of one possible SBLA (sarcoma, breast, brain, laryngeal, lung, adrenocortical cancer and leukaemia, Lynch *et al.*, 1978) family (no. 18), there were no cases of known familial cancer (multiple endocrine neoplasia, polyposis coli). Four children had neurofibromatosis (NF) with subsequent malignancy, but no parental cancer, although one mother had NF herself. One child with ataxia telangectasia developed Hodgkin's disease but there was no family history of cancer. There were 7 sets of twins (1 identical) but no sibling involvement. The median age for mothers of leukaemic children was 38.8 years, interquartile range 34.4–44.5. For mothers of children with solid tumours the median age was 37.3 years, interquartile range 32.0–41.1. For fathers of leukaemic children the median age and interquartile range were 41.7 years, and 36.4–47.6, and for fathers of children with solid tumours, 40.1 years and 35.7–44.0.

In the 638 parents (326 mothers, 312 fathers), there were 18 cases of cancer median age, 37 years, 11 of whom died of their cancer (8 mothers and 3 fathers). There were 10 deaths (2 mothers and 8 fathers) in the group without cancer, median age 47, 6 from cardiovascular disease (1 mother, 5 fathers) and 4 were accidental.

Table I gives details of parental tumours. The tumours in the mothers consisted of 8 adenocarcinomas of breast, 2 cervical squamous cancers and 1 yolk sac tumour of the ovary. All the breast cancers were of ductal type. There were no special types of breast cancer or bilateral tumours in the sample. In the fathers there were 2 adenocarcinomas of bowel (1 diagnosed at laparotomy without biopsy), 1 each of the following: cutaneous melanoma, renal adenocarcinoma, testicular teratoma, osteosarcoma and metastatic adenocarcinoma in liver of unknown primary site. There was no difference in the distribution of the childhood cancer types

Correspondence: E.N. Thompson
Received 9 April 1987; and in revised form, 5 October 1987

**Table I** Parental and child cancers

| Case | Age | Diagnosis | Outcome | Time relation of child to parental cancer | Child's diagnosis | Age of onset | Sex |
|---|---|---|---|---|---|---|---|
| *Mothers with cancer* | | | | | | | |
| 1 | 59 | Ductal adenocarcinoma of breast | A/W 2y | +13y | ALL[a] | 7y 6m | F |
| 2 | 36 | Invasive and *in-situ* ductal adenocarcinoma of breast | Died 39 | −3y | ALL | 8y 1m | M |
| 3 | 47 | Invasive and *in-situ* ductal adenocarcinoma of breast with lymph-node deposits | Died 51 | +2y | ALL | 10y 2m | M |
| 4 | 35 | Invasive and *in-situ* adenocarcinoma of breast | Died 35 | −2y | Neuroblastoma | 4y 0m | M |
| 5 | 33 | Poorly differentiated adenocarcinoma of breast | A/W 2y | +12y | Wilm's tumour | 0y 9m | F |
| 6 | 38 | Poorly differentiated adenocarcinoma of breast with lymph node deposits | Died 40 | +3y | Rhabdomyosarcoma | 1y 6m | F |
| 7 | 35 | Poorly differentiated invasive and *in-situ* ductal adenocarcinoma of breast with lymph node deposits | Died 39 | +3y | Neuroblastoma | Birth | F |
| 8 | 30 | Invasive ductal adenocarcinoma of breast | A/W 3m | +9y | Rhabdomyosarcoma | 7y 3m | M |
| 9 | 38 | *In-situ* and invasive squamous carcinoma of cervix | Died 40 | +3y | ALL | 6y 8m | M |
| 10 | 32 | Invasive squamous carcinoma of cervix | Died 34 | 0y | Astrocytoma | 2y 10m | F |
| 11 | 28 | Yolk sac tumour of ovary | Died 29 | +2y | ALL | 6y 10m | F |
| *Fathers with cancer* | | | | | | | |
| 12 | 37 | Metastatic adenocarcinoma in liver. Unknown primary | Died 37 | −7y | ALL | 10y 8m | M |
| 13 | 50 | Adenocarcinoma of caecum. Dukes grade C | A/W 1y | +4y | ALL | 9y 3m | M |
| 14 | 33 | Mixed malignant seminoma and teratoma of testis | A/W 4m | +4y | ALL | 2y 5m | F |
| 15 | 60 | Carcinoma of stomach. Operative diagnosis. No biopsy | Died 60 | 0y | ALL | 10y 10m | M |
| 16 | 30 | Polypoid malignant melanoma of skin | A/W 13y | −10y | Ewing's sarcoma | 10y 7m | F |
| 17 | 42 | Adenocarcinoma of kidney | Died 47 | −7y | Hodgkin's disease | 15y 0m | F |
| 18 | 37 | Osteosarcoma of femur | Died 38 | −5y | Hypernephroma | 13y 0m | F |

[a]Acute Lymphoblastic Leukaemia.

**Table II** Expected and actual number of parental cancers in children with malignancy

| | Type of tumour | | | | | | | | | | | | | |
|---|---|---|---|---|---|---|---|---|---|---|---|---|---|---|
| | Breast | | | | | Cervix | | | | All female tumours | | | | |
| *Mothers* | *No.* | *Exp.* | *Obs.*[a] | *RR*[b] | *P* | *Exp.* | *Obs.*[a] | *RR*[b] | *P* | *Exp.* | *Obs.*[a] | *RR*[b] | *P* |
| Leukaemics[c] | 199 | 1.39 | 3 | 2.2 | 0.16 | 0.48 | 1 | 2.1 | 0.38 | 4.41 | 5 | 1.1 | 0.45 |
| All solid tumours | 127 | 0.56 | 5 | 8.9 | 0.0003 | 0.22 | 1 | 4.5 | 0.2 | 1.90 | 6 | 3.2 | 0.01 |
| Rhabdomyosarcoma | 17 | 0.06 | 2 | 33.3 | 0.002 | 0.01 | 0 | 0 | 1.0 | 0.075 | 2 | 26.7 | 0.003 |
| Neuroblastoma | 17 | 0.044 | 2 | 45.5 | 0.001 | 0.01 | 0 | 0 | 1.0 | 0.060 | 2 | 33.3 | 0.002 |
| Total | 326 | 1.96 | 8 | 4.1 | 0.001 | 0.70 | 2 | 2.9 | 0.16 | 6.30 | 11 | 1.7 | 0.06 |

| | All male tumours | | | | |
|---|---|---|---|---|---|
| *Fathers* | *No.* | *Exp.* | *Obs.*[a] | *RR*[b] | *P* |
| Leukaemics[c] | 195 | 4.35 | 5 | 1.1 | 0.44 |
| All solid tumours | 117 | 1.87 | 2 | 1.0 | 0.56 |
| Rhabdomyosarcoma | 17 | 0.05 | 0 | 0 | 1.0 |
| Neuroblastoma | 16 | 0.04 | 0 | 0 | 1.0 |
| Total | 312 | 6.22 | 7 | 1.1 | 0.42 |

[a]Observed; [b]Relative risk; [c]Including lymphomas.

between mothers and fathers with or without cancer. There was no significant difference in the age of onset of cancer in the children whether a parent had cancer or not.

The expected and actual number of cancers found in this study with the calculated relative risks are shown in Table II. There was an increased risk of breast cancer in mothers of children with malignancy ($P=0.001$), estimated risk 4.1 times. Dividing the mothers by childhood cancer type, showed an increased risk of breast cancer in mothers of children with leukaemia and lymphoma which was not statistically significant ($P=0.16$), estimated risk 2.2 times in contrast to a highly significant ($P<0.001$) increased risk in mothers of children with solid tumours, estimated risk 8.9 times. Subdividing further by solid tumour type gives a significantly increased risk of breast cancer for mothers of children with rhabdomyosarcoma ($P=0.002$), estimated risk 33.3 times, and for mothers of children with neuroblastoma ($P=0.001$). There was no significant increase in the risk of cervical cancer. The risk of all tumours in the mothers reflected the breast cancer rate. There was no increase in cancer in the fathers.

There were two *in-situ* squamous cancers of the cervix, one benign lipoma and benign leiomyoma of the oesophagus, found in the parents, but not considered in this analysis.

### Discussion

This study has shown a significant increase of breast cancer in mothers of young children with solid tumours. In all fathers and the mothers of children with haematological malignancies the risk was not increased.

Breast cancer in mothers of 2 of the 3 children with leukaemia (only one of whom was premenopausal) were from breast cancer families, in contrast to the solid tumour group where no such history was present. Only one family (no. 18) possibly fell into the SBLA group, the child with hypernephroma, father with an osteogenic sarcoma, grandfather with unknown cancer type and a 12 year old cousin with leukaemia. A previous population based study by Birch *et al.* (1984) only looked at the families of young children with soft tissue sarcomata predominantly rhabdomyosarcoma. They found the breast cancer incidence in the mothers of these children was significantly increased with results very similar to our own. Their study was larger and so they were able to subdivide the patients further showing an even greater risk for mothers of young boys with genitourinary tumours. Only one of our cases with rhabdomyosarcoma was male with a paratesticular tumour. His mother, aged 30, developed a breast cancer 9 years after the boy's tumour had been successfully treated.

We were not able to confirm the finding of Birch *et al.* (1984) that there was any increase in bilateral and special types of breast cancer. All our tumours were unilateral, none satisfied the conventional pathological criteria for any of the special types of breast cancer. This might be due to the fact that our numbers are small and only two of the five young children with solid tumour had rhabdomyosarcoma.

There are many factors which are known to increase the risk of breast cancer, increased age at first pregnancy and family history of breast cancer being the most prominent. Although we did not study the former variable in detail, the age at first pregnancy for the breast cancer mothers did not appear to be increased. We did not undertake a detailed pedigree family history on previous generations of these families, but knew that in two instances, maternal sisters or mothers also had a history of breast cancer. These were the two older children with leukaemia.

There is always a danger in using population-based morbidity data as a control, as many uncontrolled variables are present, such as family history of breast cancer, age of the mother at first pregnancy, domicile, smoking habits, all of which may increase the risks of cancer. Because the study was retrospective, there was always the danger that it is easier to find the data on unusual cases, such as parents with cancer, and so bias the figures towards an increased risk. It is also possible that cancers may have been missed, because if one parent died there would be a greater chance of the family moving out of the area. The best way of overcoming these problems would be to investigate a case controlled study matching the known variables affecting breast cancer. There are many theories which can account for our findings, which may have a heritable component, or an environmental factor affecting mother and child. Further genetic studies are needed to clarify these points. However, we feel that the risk of maternal cancer, particularly breast cancer, is higher than expected in mothers of young children with solid tumours, but not leukaemia, which is a much commoner childhood cancer. Such cancers may present years after the child's illness. The need to study the mothers of these children prospectively is important to determine whether counselling or regular surveillance monitoring of these mothers is justified or not.

### References

BIRCH, J.M., HARTLEY, A.L., MARSDEN, H.B., HARRIS, M. & SWINDELL, R. (1984). Excess risk of breast cancer in mothers of children with soft-tissue sarcomas. *Br. J. Cancer*, **49**, 325.

DRAPER, G.J., HEAF, M.M. & KINNIER WILSON, L.M. (1977). Occurrence of childhood cancers among sibs and estimation of familial risks. *J. Med. Genet.*, **14**, 81.

FRAUMENI, J.F. JNR. & GLASS, A.G. (1968). Wilm's tumour and congenital aniridia. *JAMA*, **206**, 825.

JENSEN, R.D. & MILLER, R.W. (1971). Retinoblastoma: Epidemiologic characteristics. *N. Engl. J. Med.*, **285**, 307.

KRAMER, S., MEADOWS, A.T., JARRETT, P. & EVANS, A.E. (1983). Incidence of childhood cancers: Experience of a decade in a population based registry. *J. Natl Cancer Inst.*, **70**, 49.

LI, F.P. (1978). Host factors in the development of childhood cancers. *Semin. Oncol.*, **5**, 17.

LI, F.P. & FRAUMENI, J.F. JNR. (1969). Soft tissue sarcomas, breast cancer and other neoplasms. A familial syndrome? *Ann. Int. Med.*, **71**, 747.

LI, F.P. & FRAUMENI, J.F. JNR. (1982). Prospective study of a family cancer syndrome. *JAMA*, **247**, 2692.

LYNCH, H.T., MULCAHY, G.M., HARRIS, R.E., GUIRGIS, H.A. & LYNCH, J.F. (1978). Genetic and pathologic findings in a kindred with hereditary sarcoma, breast cancer, cancer, brain tumours, leukaemia, lung, laryngeal and adrenal cortical carcinoma. *Cancer*, **41**, 2055.

WATERHOUSE, J., MUIR, C., SHANMUGARTNAM, K. & POWELL, J. (eds) (1982). *Cancer incidence in five continents*. IARC Scientific Publications, **42**, IARC: Lyon, 552.

ZAR, J.H. (1974). *Biostatistical Analysis*, 2nd ed., p.412. Prentice Hall Inc.: Hemel Hempstead, Herts.